# P215: Tracking infections in outpatient hemodialysis unit in Saudi Arabia

**DOI:** 10.1186/2047-2994-2-S1-P215

**Published:** 2013-06-20

**Authors:** N Dagunton, A El-Saed, A Al Sayyari, F Hejaili, A Azzam, M Alarcon, H Balkhy

**Affiliations:** 1 Infection Prevention & Control, King Abdulaziz Medical City, Riyadh 11426, Saudi Arabia; 2 Department of Nephrology, King Abdulaziz Medical City, Riyadh 11426, Saudi Arabia; 3 Hemodialysis Program, King Abdulaziz Medical City, Riyadh 11426, Saudi Arabia

## Introduction

Bacteremias and localized infections of the vascular access site are associated with high morbidity and mortality among end-satge renal (ESRD) patients.

## Objectives

At King Abdulaziz Medical City (KAMC) in Riyadh (SA), surveillance among ESRD patients was conducted to detect trends in dialysis related infections and evaluate effectiveness of prevention measures.

## Methods

Following the methodology set by the National Healthcare Safety Network (NHSN), a 32-month prospective surveillance (monthly average of 218 patients-months) was conducted from 2008 to 2012 at the Outpatient Hemodialysis Unit at KAMC. Patients were monitored for any of the three dialysis events; outpatient start of an intravenous (IV) antimicrobial, evidence of local access site infection (LASI) and access-associated bacteremia.

Infection control measures were instituted throughout the whole period of study but stringent ones were strictly emphasized starting early 2012. Interventions implemented included the use of Chlorhexidine impregnated transparent dressings, strict monitoring of aseptic dressing technique, provision of an Arabic educational brochure for patient education, provision of an alcohol hand gel in each patient trolleys and judicious use of antibiotics.

## Results

The combined rates of all three types of events significantly declined from 19.2 to 7.6 per 100 patients-months which constituted about 60% reduction. Comparing the rates between patients with AV fistulas and patients with permanent catheter , there was an 85% reduction in rates among patients with AV fistula (from 2.6 to .4) and 54% decline in patients with permanent catheter (from 15.6 to 7.2). The rate of access-associated bacteremia among patients with AV fistula dropped from .5 to zero while the rate of patients with permanent catheter was reduced from 3.8 to 1.9.

## Conclusion

Tracking dialysis events and following basic infection control measures are indeed vital in the prevention of infections among ESRD patients. The surveillance has shown a significant decline in both AV fistula and permanent catheter but patients with AV fistula better responded to preventive measures.

## Disclosure of interest

None declared

